# Phacoemulsification with goniosynechialysis for nanophthalmos with secondary angle-closure or secondary angle-closure glaucoma: a 20-case series and stepwise management strategy

**DOI:** 10.1186/s12886-026-04942-2

**Published:** 2026-06-01

**Authors:** Hongyan Zhu, Zuohong Wu, Yong Wang, Xun Zhan, Jin Lv, Li Ye

**Affiliations:** Aier Eye Hospital of Wuhan University, No. 481 Zhongshan Road, Wuchang District, Wuhan City, 430064 P. R. China

**Keywords:** Nanophthalmos, Secondary angle-closure glaucoma, Goniosynechialysis, Phacoemulsification, Stepwise management, Axial length

## Abstract

**Background:**

Nanophthalmos is a rare congenital ocular disorder characterized by a short axial length (AL), which predisposes to secondary angle-closure and secondary angle-closure glaucoma. Surgical management is challenging due to high complication risks. This study aimed to evaluate the feasibility and efficacy of phacoemulsification with intraocular lens implantation and goniosynechialysis (Phaco + IOL+GSL) in nanophthalmos with secondary angle-closure or secondary angle-closure glaucoma, and to propose a stepwise complication management strategy.

**Methods:**

This retrospective case series included 20 eyes of 20 patients diagnosed with nanophthalmos (AL < 20 mm) and PAC (2 eyes) or ACG (18 eyes) who underwent Phaco + IOL+GSL between January 2022 and Jun 2025. Patients were divided into three groups based on AL: Group A (14 mm ≤ AL < 16 mm, 9 eyes), Group B (16 mm ≤ AL < 18 mm, 4 eyes), and Group C (18 mm ≤ AL < 20 mm, 7 eyes). Preoperative and postoperative uncorrected visual acuity (UCVA, logMAR) and best-corrected visual acuity (BCVA, logMAR) were recorded, intraocular pressure (IOP), number of antiglaucoma medications, and intraoperative and postoperative complications were analyzed.

**Results:**

At the final follow-up (6–12 months postoperatively), the median IOP significantly decreased from 25.5 [16.0, 35.0] mmHg to 16.0 [14.0, 19.0] mmHg (*P* < 0.05). The median number of antiglaucoma medications decreased from 2.5 [0.0, 4.0] to 0.0 [0.0, 0.0] (*P* < 0.05). UCVA improved from 1.30 [1.00, 1.70] to 0.40 [0.12, 1.10] (*P* < 0.05), and BCVA improved from 0.82 [0.40, 1.30] to 0.50 [0.30, 0.90]** (***P* < 0.05**).**The qualified success rate was 95.0% (19/20), and the complete success rate was 80.0% (16/20). The intraoperative complication rate was 25.0% (5/20), primarily shallow anterior chamber. The postoperative complication rate was 40.0% (8/20), primarily shallow anterior chamber with elevated IOP. Complications were concentrated in Groups A and B. A stepwise management strategy—initiating with medical/laser therapy, escalating to cyclophotocoagulation, and reserving pars plana vitrectomy for refractory cases—achieved successful complication management.

**Conclusion:**

For nanophthalmos with secondary angle-closure glaucoma, Phaco + IOL+GSL appears to be a feasible and effective procedure, particularly when combined with a stepwise complication management strategy. It may serve as an initial surgical option before resorting to more invasive combined procedures. However, the high complication rate in eyes with AL < 16 mm warrants extreme caution and readiness for escalation. The retrospective design and small sample size limit the generalizability of our findings; larger prospective studies are needed to confirm these results.

## Background

Nanophthalmos is a rare congenital ocular maldevelopment characterized by a significantly shortened axial length (AL), thickened sclera, high hyperopia, and a shallow anterior chamber [1]. Primary angle closure (PAC) and secondary angle-closure glaucoma (ACG) are among its most serious complications, and its management is challenging [2, 3]. Due to crowded anterior segments and unique scleral properties, patients with nanophthalmos are at high risk for serious complications such as choroidal effusion, malignant glaucoma, and expulsive suprachoroidal hemorrhage during traditional filtering surgeries [4]. Yalvac et al. [5] reported that the incidence of choroidal detachment after filtering surgery was 25%, and uveal effusion occurred in 50% of cases. These findings were further corroborated by Rajendrababu et al. [3].

Given the anatomical peculiarities and risks, many surgeons have explored cyclophotocoagulation as an alternative [6, 7]. Abdelrahman et al. [6] demonstrated that transscleral cyclophotocoagulation (TSCPC) significantly reduced IOP over 6 months without severe complications like hypotony or choroidal effusion. However, as a destructive procedure, the long-term efficacy and potential complications of cyclophotocoagulation warrant cautious evaluation.

Recently, phacoemulsification with intraocular lens implantation (Phaco + IOL) has been increasingly used in nanophthalmic eyes, and its safety profile has been preliminarily recognized [8]. For cases complicated by PAC or ACG, most surgeons adopt combined procedures, often incorporating anterior vitrectomy and/or sclerectomy. Studies by Wei et al. [9] and Fan et al. [10] indicated that combined surgeries yielded better IOP control, fewer complications, and higher success rates compared to filtering surgery alone. Anterior vitrectomy has been shown to reduce the risk of postoperative malignant glaucoma [10, 11], while prophylactic sclerectomy helps prevent uveal effusion [10, 11]. However, both vitrectomy and sclerectomy increase surgical trauma and operating time, presenting additional challenges for patients.

Phacoemulsification combined with IOL implantation and goniosynechialysis (Phaco + IOL+GSL) has gained widespread use in managing PAC and ACG, particularly with coexisting cataract, and its efficacy and safety have been supported by numerous studies [12, 13]. The mechanism primarily involves removing the relatively thick lens to deepen the anterior chamber, thereby alleviating pupillary block and peripheral anterior synechiae (PAS), while gonioscopic separation of synechiae aims to reopen the physiological aqueous outflow pathway via the trabecular meshwork [14]. Compared to complex combined surgeries, Phaco + IOL+GSL offers the advantages of a simpler technique and shorter operative time.

Therefore, this retrospective study aims to evaluate the feasibility and efficacy of Phaco + IOL+GSL for nanophthalmos with secondary angle-closure or secondary angle-closure glaucoma, and to explore influencing factors. Compared with more complex combined procedures (e.g., with prophylactic vitrectomy or sclerectomy) reported in the literature [9, 10], this technique is simpler and less invasive. We also propose a stepwise management strategy for postoperative complications.

## Methods

### Study population

This retrospective case series consecutively enrolled 20 eyes of 20 patients diagnosed with nanophthalmos (axial length < 20 mm) and secondary angle-closure who underwent Phaco + IOL+GSL at Aier Eye Hospital of Wuhan University between January 2022 and June 2025. Of these, 18 eyes had glaucomatous optic neuropathy (secondary angle-closure glaucoma) and 2 eyes had angle closure without glaucomatous damage (secondary angle-closure). The study adhered to the tenets of the Declaration of Helsinki and was approved by the hospital’s Ethics Committee. Informed consent was obtained from all patients.

1. Inclusion Criteria: (1) AL < 20 mm measured by IOLMaster-700; (2) Gonioscopy confirming angle closure ≥ 180° (defined as failure to visualize the pigmented posterior trabecular meshwork under dynamic gonioscopy, supplemented by ultrasound biomicroscopy); (3) Postoperative follow-up ≥ 6 months.
2. Exclusion Criteria: (1) Previous intraocular surgery; (2) Coexisting ocular diseases such as uveitis, ocular trauma, or retinal detachment; (3) Incomplete clinical data.

### Grouping and methods

Patients were divided into three groups based on AL: Group A (14 mm ≤ AL < 16 mm, 9 eyes); Group B (16 mm ≤ AL < 18 mm, 4 eyes); Group C (18 mm ≤ AL < 20 mm, 7 eyes). Baseline characteristics were collected and compared among the groups.

All 20 eyes underwent both IOLMaster 700 (swept-source OCT biometry) and supine UBM (50 MHz) examination. Corrected ACD was calculated as IOLMaster-ACD minus central corneal thickness (CCT) in eyes where IOLMaster successfully acquired measurements. In the 2 eyes where IOLMaster failed due to extremely shallow anterior chamber or dense opacity, ACD measured by UBM was used [15]. 

### Surgical technique

All surgeries were performed by two experienced glaucoma and cataract surgeons.

1. Preoperative Preparation: Topical IOP-lowering medications (excluding prostaglandin analogs) were administered 1–3 days preoperatively. Intravenous 20% mannitol (250 ml) was administered on the day of surgery, 30 min before the procedure. For patients with preoperative IOP ≥ 30 mmHg despite triple topical therapy, after excluding contraindications (renal impairment, nephrolithiasis, sulfonamide allergy), oral acetazolamide 25 mg once or twice daily was administered 1–3 days preoperatively.
2. Surgical Equipment: Two Alcon phacoemulsification systems (Centurion and Infinity) were used alternately.Goniosynechialysis was performed using a 360° rotatable direct gonioscopic lens (Ahmed DVX Surgical Gonio Lens, Ocular Instruments).
3. Surgical Procedure: Anesthesia: For patients with AL < 16 mm, combined general and retrobulbar anesthesia was used with epinephrine added to the retrobulbar injectate (0.01 mL of 1:1,000 epinephrine in 5 mL of 2% lidocaine, injected volume 2 mL). For patients with AL ≥ 16 mm, either general anesthesia or retrobulbar anesthesia was used; when retrobulbar anesthesia was chosen, epinephrine was added as above. This strategy aimed to reduce choroidal congestion in nanophthalmic eyes.

After induction of anesthesia but before any corneal incision, if the anterior chamber (AC) was too shallow to safely perform corneal incisions, we performed a vitreous tap using a 1 mL syringe with a 26G needle at the pars plana (3.5 mm from the limbus). Aspiration of 1–2 times removed typically ≤ 0.1 mL of liquefied vitreous, resulting in immediate AC deepening.

A 2.2 mm main incision and a side port were created at the limbus. Continuous curvilinear capsulorhexis was performed, followed by hydrodissection and phacoemulsification of the nucleus and aspiration of the residual cortex. During phacoemulsification, to maintain AC stability, viscoelastic was injected through the side port before withdrawing the phaco tip when switching between ultrasound and I/A.

Single IOL was implanted in 16 eyes (single-piece, hydrophobic acrylic IOL). Two eyes aiming for emmetropia underwent piggyback IOL implantation (one three-piece IOL in the ciliary sulcus and one single-piece IOL in the bag). Two eyes with preoperative vision of light perception or no light perception did not receive an IOL.

Prophylactic peripheral iridectomy (PI) was performed in eyes meeting any of the following criteria: (1) axial length < 16 mm; (2) preoperative IOP ≥ 30 mmHg despite maximal medical therapy; or (3) a recent acute angle-closure attack. In eyes with PI, the IOL haptics were positioned away from the PI site.

Subsequently, under direct gonioscopic view, extensive blunt separation of PAS was performed using an iris repositor, aiming to maximally re-expose the functional trabecular meshwork and ciliary body band, targeting a separation extent of ≥ 270°.

Four eyes developed intraoperative shallow anterior chamber/aqueous misdirection. In 2 eyes, anterior chamber depth was restored via pars plana vitreous cavity tap (23G needle, aspirating 0-0.1 ml fluid), allowing surgery to proceed. The other 2 eyes with persistent shallow anterior chamber underwent anterior vitrectomy via the pars plana; one of these also had posterior capsular rupture requiring IOL scleral fixation. Significant hemorrhage during synechialysis in one eye was managed by injecting a little air bubble into the anterior chamber.

### Postoperative management and follow-up

Postoperative topical antibiotics, corticosteroids, and nonsteroidal anti-inflammatory drugs were routinely administered and tapered. IOP, anterior chamber depth, inflammation, and fundus were closely monitored. If shallow anterior chamber or elevated IOP occurred, intravenous mannitol and intensified anti-inflammatory therapy were initiated, cycloplegics (atropine 1% twice daily) were also used. If medical therapy is ineffective within 24–48 h, Nd: YAG laser peripheral anterior capsulotomy (peripheral to the IOL optic zone) combined with posterior capsulotomy and anterior hyaloidotomy were performed via the existing PI site or at the IOL edge [16]. If laser access was unavailable or ineffective, cyclophotocoagulation or secondary surgery (anterior vitrectomy + goniosynechialysis ± IOL explantation) was considered.

### Observation indicators

Preoperative and postoperative UCVA (logMAR), BCVA (logMAR), IOP, number of antiglaucoma medications, and intraoperative and postoperative complications were recorded.

Surgical Success Criteria: Complete success: IOP ≤ 21 mmHg without antiglaucoma medications; Qualified success: IOP ≤ 21 mmHg with medications.

### Statistical analysis

SPSS 26.0 was used for analysis. Visual acuities were converted to logMAR (counting fingers, hand motions, light perception, and no light perception were assigned values of 2.0, 2.3, 2.7, and 3.0, respectively [17]. Normally distributed continuous data are presented as mean ± standard deviation, and non-normally distributed data as median [Q1, Q₃]. The Wilcoxon signed-rank test or Kruskal-Wallis H test was used for comparisons. Categorical data are presented as n (%), compared using Fisher’s exact test. Multiple comparisons employed Bonferroni correction. *P* < 0.05 was considered statistically significant.

## Results

### Patient baseline characteristics

Twenty eyes of 20 patients were included (8 males [40.0%], 12 females [60.0%]). No significant differences were found among the three groups regarding age, gender, preoperative IOP, number of preoperative medications, or preoperative visual acuity (*P* > 0.05), indicating comparability (Table 1).

**Table 1:** Comparison of baseline characteristics among the three groups

Indicator	Group A (14–16 mm, *n* = 9)	Group B (16–18 mm, *n* = 4)	Group C (18–20 mm, *n* = 7)	*P*-value	Statistical Test
Age (years), M [Q1, Q3]	55.0 [48.0, 59.0]	56.5 [44.3, 68.0]	59.0 [51.5, 73.0]	0.770	Kruskal-Wallis
Gender (Male), n (%)	4 (44.4%)	2 (50.0%)	2 (28.6%)	0.804	Fisher’s Exact
Preop IOP (mmHg), M [Q1, Q3]	21.0 [16.5, 33.5]	29.5 [18.8, 40.5]	34.0 [17.0, 54.0]	0.233	Kruskal-Wallis
Preop Meds (n), M [Q1, Q3]	3.0 [0.0, 4.0]	3.0 [3.0, 3.0]	1.0 [0.0, 3.0]	0.056	Kruskal-Wallis
Preop UCVA (logMAR), M [Q1, Q3]	1.40 [1.22, 1.70]	1.40 [1.03, 1.78]	1.10 [0.96, 2.20]	0.924	Kruskal-Wallis
Preop BCVA (logMAR), M [Q1, Q3]	1.00 [0.82, 1.55]	1.20 [0.88, 1.48]	0.70 [0.40, 2.00]	0.660	Kruskal-Wallis
Preop ACD	1.26 [0.92, 1.56]	1.38 [1.14, 2.38]	1.68 [1.27, 1.71]	0.388	Kruskal-Wallis

Note: M [Q1, Q3]: Median [First Quartile, Third Quartile].Preoperative corrected anterior chamber depth (ACD) measured by IOLMaster 700 or supine UBM (see Methods)

Among the 20 enrolled eyes, 2 eyes (10.0%) had angle closure without glaucomatous damage (PAS ≥ 180°,no optic nerve damage and no reliable visual field defect). The remaining 18 eyes (90.0%) met the criteria for secondary angle-closure glaucoma (PAS ≥ 180°, IOP > 21 mmHg, and documented glaucomatous optic neuropathy). In this glaucomatous subgroup, the median cup-to-disc ratio was 0.75 [0.60, 0.90]. Reliable Humphrey visual field (24 − 2 SITA-Standard) was obtained in 8 of these 18 eyes (44.4%); median mean deviation (MD) was − 15.0 dB (range − 32.5 to − 7.0 dB). Using the Hodapp-Parrish-Anderson classification, 5 eyes were graded as moderate glaucoma (MD − 6 to − 12 dB) and 3 eyes as advanced glaucoma (MD < − 12 dB).

### Surgical efficacy evaluation

The median preoperative IOP was 25.5 [16.0, 35.0] mmHg, which decreased to 16.0 [14.0, 20.0] mmHg at 3 months postoperatively (*P* < 0.05) and at the 6–12 month follow-up, it was 16.0 [12.0, 19.0] mmHg, indicating a significant and sustained IOP reduction (Table 2).

The median number of antiglaucoma medications decreased from 2.5 [0.0, 4.0] preoperatively to 0.0 [0.0, 0.0] at 3 months postoperatively (*P* = 0.003) and remained at 0.0 [0.0, 0.0] at the 6–12 month follow-up, with no statistically significant difference compared to the 3-month postoperative visit (*P* = 1.000), demonstrating that the surgery significantly reduces medication dependence, and this effect is sustained over time (Table 2).

UCVA (logMAR) improved from 1.30 [1.00, 1.70] preoperatively to 0.40 [0.12, 1.10] postoperatively (*P* < 0.001). BCVA (logMAR) improved from 0.82 [0.40, 1.30] to 0.50 [0.30, 0.90] (*P* = 0.012) (Table 3).

At the final follow-up (6–12 months), the qualified success rate was 95.0% (19/20), the complete success rate was 80.0% (16/20), and the failure rate was 5.0% (1/20).

**Table 2:** Changes in IOP and number of antiglaucoma medications

Indicator	Preoperative	Postop 3 Months	Postop 6–12 Months	*P*^1^-value (Pre vs. postop 3 M)	*P*^1^-value (Pre vs. 6-12 M)	*P*^2^-value (3 M vs. 6-12 M)
IOP (mmHg), M [Q1, Q3]	25.5[16.0,35.0]	16.0 [14.0,20.0]	16.0[14.0,19.0]	0.004	0.008	1.000
Meds (n), M [Q1, Q3]	2.5[0.0,4.0]	0.0[0.0,0.0]	0.0[0.0,0.0]	0.003	0.002	1.000

Note: Wilcoxon signed-rank test with Bonferroni correction. (For the P^1^ values, a total of three comparisons were made, and the corrected significance level was set at 0.05/3 = 0.017. A corrected P-value < 0.017 was considered statistically significant. For the P^2^ values, two comparisons were made, with a corrected significance level of 0.05/2 = 0.025. A P-value > 0.025 indicated no statistically significant difference)

**Table 3:** Changes in UCVA and BCVA (logMAR)

Visual Acuity (logMAR)	Preoperative	Postoperative	*P*-value
UCVA (*n* = 20)	1.30 [1.00, 1.70]	0.40 [0.12, 1.10]	< 0.001
BCVA (*n* = 20)	0.82[0.40, 1.30]	0.50 [0.30, 0.90]	0.012

Note: Wilcoxon signed-rank test

Intergroup comparison of surgical outcomes (Table 4) showed that: In Group A, both postoperative intraocular pressure (IOP) and the number of medications decreased significantly compared to preoperative levels (*P* < 0.05). In Group C, there was no statistically significant difference in the number of medications, but postoperative IOP was significantly lower than preoperative IOP (*P* < 0.05). Group B exhibited the largest magnitude of change in both IOP and medication number; however, none of the P values reached statistical significance, suggesting that a larger sample size may be needed to confirm the statistical significance of this effect.

**Table 4:** Preoperative and postoperative changes in IOP and medications by group

Group	Preop IOP (mmHg), M [Q1,Q3]	Final IOP (mmHg), M [Q1,Q3]	*P*-value (IOP)	Preop Meds (*n*), M [Q1,Q3]	Final Meds (*n*), M [Q1,Q3]	*P*-value (Meds)
A	21.0 [16.5, 33.5]	18.0 [17.0, 19.0]	0.012	3.0 [0.0, 4.0]	0.0 [0.0, 1.0]	0.020
B	29.5 [18.8, 40.5]	14.5 [12.0, 15.0]	0.250	3.0 [3.0, 3.0]	0.0 [0.0, 2.0]	0.250
C	34.0 [17.0,54.0]	13.0[12.0,16.0]	0.031	1.0 [0.0, 3.0]	0.0 [0.0, 0.0]	0.125

Note: Wilcoxon signed-rank test for within-group changes

### Safety evaluation: Complications and management

1. **Intraoperative Complications**: The overall intraoperative complication rate was 25.0% (5/20). Shallow anterior chamber/aqueous misdirection was the most common complication (4 eyes, 20.0%), with one eye concurrently experiencing posterior capsular rupture (PCR rate 5.0%). No expulsive suprachoroidal hemorrhage occurred. Complications were concentrated in Groups A and B, with no complications in Group C (Table 5). The difference in the incidence of any intraoperative complication among groups (Group A 33.3% vs. Group B 50.0% vs. Group C 0.0%) was statistically significant (*P* = 0.044, Fisher’s exact test).The recorded intraoperative shallow AC/aqueous misdirection rate (4/20, 20.0%) does not include cases that were successfully managed by the pre-surgical vitreous tap procedure (performed before any corneal incision), as that maneuver is a proactive pre-surgical technique and not considered an intraoperative complication [18, 19]. 

**Management**: All 4 eyes with intraoperative aqueous misdirection/shallow anterior chamber underwent pars plana vitreous tap (23G needle, aspirating approximately 0-0.1 ml). This restored anterior chamber depth in 2 eyes, allowing surgery to continue. The other 2 eyes required additional anterior vitrectomy; one of these also had PCR requiring IOL scleral fixation. One eye with significant hemorrhage during synechialysis was managed by anterior chamber air injection.

**Table 5:** Intraoperative complications by axial length group [n(%)]

Group	Total Eyes	Shallow AC	PCR	Hyphema	Any Complication*	Intergroup *P*-value
A(14–16 mm)	9	2 (22.2%)	0 (0.0%)	1 (11.1%)	3 (33.3%)	
B(16–18 mm)	4	2 (50.0%)	1 (25.0%)	0 (0.0%)	2 (50.0%)	
C(18–20 mm)	7	0 (0.0%)	0 (0.0%)	0 (0.0%)	0 (0.0%)	
Total	**20**	**4 (20.0%)**	**1 (5.0%)**	**1(5.0%)**	**5 (25.0%)**	**0.044**

Note: PCR: Posterior Capsular Rupture. *"Any Complication” refers to independent eyes experiencing at least one complication

**2. Postoperative Complications**: Seven complications occurred within the first week, and one at 4 weeks postoperatively, with a total rate of 40.0% (8/20). The main manifestation was shallow anterior chamber with elevated IOP (25.0%, 5/20), followed by isolated shallow anterior chamber (5.0%, 1 eye), isolated elevated IOP (5.0%, 1 eye), and choroidal detachment (5.0%, 1 eye). All complications occurred in Groups A and B. Group A had the highest incidence (66.7%). The intergroup difference was statistically significant (*P* = 0.008, Fisher’s exact test) (Table 6). Pairwise comparisons showed Group A (shortest AL) had a significantly higher complication rate than Group C (longest AL) (*P* = 0.009). Group B also had a high rate (50.0%), but its comparison with Group C did not reach the Bonferroni-corrected significance level (*P* < 0.017, α = 0.05/3 ≈ 0.017), possibly due to its small sample size (*n* = 4).

**Table 6:** Postoperative complications by axial length group [n(%)]

Group	Total Eyes	Shallow AC	High IOP	Shallow AC + High IOP	Choroidal Detachment	Any Complication	Intergroup *P*-value
A(14–16 mm)	9	1(11.1%)	1(11.1%)	4 (44.4%)	0 (0.0%)	6 (66.7%)	
B(16–18 mm)	4	0 (0.0%)	0 (0.0%)	1 (25.0%)	1 (25.0%)	2 (50.0%)	
C(18–20 mm)	7	0 (0.0%)	0 (0.0%)	0 (0.0%)	0 (0.0%)	0 (0.0%)	
Total	**20**	**1 (5.0%)**	**1 (5.0%)**	**5 (25.0%)**	**1 (5.0%)**	**8 (40.0%)**	**0.008**

Note: Fisher’s exact test

**3. Management of Postoperative Complications**: Among the 8 eyes with complications, 1 eye with choroidal detachment resolved with conservative medical treatment. The remaining 7 eyes (including 2 with piggyback IOLs) presented with shallow AC and/or elevated IOP. In these 7 eyes, initial intervention with laser peripheral anterior capsulotomy (peripheral to IOL optic zone) combined with posterior capsulotomy and anterior hyaloidotomy were performed in 3 eyes, successfully resolving shallow AC in 2 eyes; the other eye was unresponsive and subsequently underwent successful cyclophotocoagulation for IOP control. Management and outcomes for the other 4 eyes were as follows: Among 2 eyes with single IOL implantation, cyclophotocoagulation was ineffective in both. One eye underwent successful secondary surgery (anterior vitrectomy + goniosynechialysis) at 1 month, while the other declined further intervention and had uncontrolled IOP at final follow-up, with vision declining from hand motions to light perception. Among the 2 eyes with piggyback IOLs, one underwent secondary surgery (anterior vitrectomy + goniosynechialysis) 2 months after failed cyclophotocoagulation. The other eye developed early malignant glaucoma with severe exudation, requiring emergency secondary surgery (anterior vitrectomy + sulcus IOL removal + goniosynechialysis) on postoperative day 3, which successfully controlled IOP and exudation.

As shown in Table 7, secondary surgery (vitrectomy combined with goniosynechialysis ± IOL removal) had a 100% success rate (3/3). Nd: YAG laser posterior capsulotomy was 66.7% effective (2/3). Conventional cyclophotocoagulation was 25.0% effective (1/4).

**Table 7:** Efficacy of different complication management strategies

Management Strategy	Eyes Treated (*n*)	Effective (*n*)	New Complications (*n*)	Success Rate
Nd: YAG *	3	2	0	66.7%
Cyclophotocoagulation	4	1	0	25.0%
Secondary Surgery (PPV + GSL±IOL)	3	3	0	100%

Note: *laser peripheral anterior capsulotomy (peripheral to IOL optic zone) combined with posterior capsulotomy and anterior hyaloidotomy [16].

**4. Postoperative Refractive Status in Single IOL Implantation (Group A)**: Analysis of 5 eyes in Group A (AL < 16 mm) that received single IOL implantation showed persistent significant hyperopia postoperatively (mean SE + 13.45 D). Comparison of the difference between preoperative and postoperative SE revealed minimal change in residual refractive error with single IOL implantation (mean difference + 0.80 ± 1.48 D) (Table 8).

**Table 8:** Refractive status changes in 5 eyes with single IOL implantation in group A (14–16 mm)

Case	Preop SE (D)	Postop SE (D)	Difference (Postop-Preop) (D)
Tan XX	+ 12.75	+ 13.50	+ 0.75
Liao XX	+ 14.00	+ 13.00	-1.00
Sun X	+ 16.50	+ 16.50	0.00
Wang XX	+ 11.00	+ 12.00	+ 1.00
Liu XX	+ 9.00	+ 12.25	+ 3.25
Mean	+ 12.65	+ 13.45	+ 0.80 ± 1.48

## Discussion

Surgical management of nanophthalmos with secondary angle-closure glaucoma is challenging due to the high risk of complications. Unlike prior studies that emphasized combined vitrectomy or sclerectomy upfront, our study’s main contributions are: (1) detailed technical refinements for the extreme AL subgroup (< 16 mm); (2) a stepwise management algorithm for postoperative complications derived from our retrospective experience; and (3) caution against routine piggyback IOL implantation in nanophthalmos.

Technical refinements for AL < 16 mm included: anesthesia with low-dose epinephrine to reduce choroidal congestion; proactive pre-surgical vitreous tap to deepen the shallow AC; prophylactic PI as a laser access port; and avoidance of piggyback IOLs based on our two cases that both developed malignant glaucoma.

It is well established that lens extraction (phacoemulsification) alone can deepen the anterior chamber, widen the angle, and reduce intraocular pressure (IOP) in nanophthalmic eyes with angle closure [4, 9, 10]. However, studies on primary angle-closure disease have shown that the IOP reduction after phacoemulsification alone is limited, particularly in eyes with extensive peripheral anterior synechiae (PAS) or higher baseline IOP [20]. A meta-analysis of nine randomized controlled trials reported that phacoemulsification combined with goniosynechialysis (GSL) achieved greater IOP reduction compared with phacoemulsification alone in patients with angle-closure disease (mean difference, 1.81 mmHg; *p* = 0.002) [20]. In the present series, the baseline IOP was relatively high (median 25.5 mmHg) and PAS was extensive (≥ 180°). Therefore, the additional IOP reduction associated with GSL may be relevant for this specific population.

Our final follow-up showed a qualified success rate of 95.0% and a complete success rate of 80.0%. This efficacy is comparable to the 71.4% complete success rate reported by Zhang et al. [21] for 23G pars plana vitrectomy combined with lensectomy (without IOL implantation). Notably, Wei et al. [9] employed a more complex procedure (phacoemulsification combined with anterior vitrectomy and posterior capsulotomy), reporting an overall success rate of 88.9%, but a lower complete success rate (42.5%) than our study. This discrepancy may be attributed to the absence of gonioscopic synechialysis, potentially affecting the completeness of angle opening. However, their very low complication rate (4.4%) also underscores the value of anterior vitrectomy in relieving ciliary block and managing high-risk factors.

The crucial question is whether such prophylactic procedures should be routinely combined during initial surgery. The randomized controlled trial by Rajendrababu et al. [22] provides key insights. They found no statistical difference in complication risk between “phacoemulsification with prophylactic sclerostomy” and “phacoemulsification alone,” with the combined procedure inducing significant inflammation. However, they observed a trend towards lower complication rates and suggested considering combined surgery. This reveals the clinical dilemma: the potential reduction in risk must be balanced against the greater trauma and inflammatory burden imposed on all patients by a more complex procedure.

In this context, our proposed stepwise treatment strategy offers a more targeted solution. We do not negate the role of vitrectomy/sclerostomy but reserve them as “backup plans.” We primarily selected the minimally invasive Phaco + IOL+GSL, enabling 90.0% (18/20) of patients to complete surgery safely. Anterior vitrectomy was added only for the minority of high-risk patients (10.0%, 2/20) with persistent intraoperative shallow AC/aqueous misdirection. Another subset (15.0%, 3/20) developed postoperative malignant glaucoma refractory to medical and laser therapy, ultimately requiring secondary pars plana vitrectomy (PPV) to resolve the shallow anterior chamber and control IOP. It should be noted that one of these three cases requiring secondary surgery was the patient who had already received intraoperative vitrectomy. Therefore, overall, 4 out of 20 patients (20.0%) eventually required vitrectomy (either during initial surgery or postoperatively) for complication management.

This outcome supports the rationale for the stepwise strategy on two levels: First, it allowed 80.0% (16/20) of patients to avoid vitrectomy during initial surgery, with no cases of severe complications like expulsive suprachoroidal hemorrhage, thereby upholding the principle of minimal invasiveness. Second, by prioritizing Phaco + IOL+GSL, combining anterior vitrectomy intraoperatively as needed (10% in this study), and aggressively performing pars plana vitrectomy for refractory complications unresponsive to laser and cyclophotocoagulation, a high final success rate (95.0% in this study) was achieved.This stepwise management strategy was developed retrospectively; it is not a validated prospective algorithm. Readers should interpret it as a conceptual framework.

IOP control in this study was achieved through thorough goniosynechialysis performed under direct gonioscopic view. While the contribution of GSL versus lens extraction alone cannot be fully isolated, the meta-analysis evidence [20] supports the incremental benefit of adding GSL, especially in eyes with high baseline IOP and extensive PAS. This aligns with the concept of internal drainage procedures centered on Phaco + GSL, which has been increasingly adopted in the general ACG population [23]. Tang Yihua et al. [12] emphasized that gonioscopy-guided synechialysis with confirmation of angle opening is crucial for achieving better IOP-lowering outcomes. All cases in our study achieved synechialysis over ≥ 270°, aiming to expose the functional trabecular meshwork, which establishes a solid anatomical foundation for long-term IOP control [12, 13, 24].We did not routinely combine goniotomy because published evidence suggests that goniotomy increases the risk of ciliochoroidal detachment (approximately 20% in non-nanophthalmic eyes) without proven additional IOP-lowering benefit over GSL alone in angle-closure glaucoma [25, 26]. 

Visual improvement primarily stemmed from clearing the optical media and addressing the pre-existing hyperopia. Surgery significantly improved both UCVA (median logMAR from 1.30 to 0.40, *p* < 0.001) and BCVA (median logMAR from 0.82 to 0.50 , *p* =0.012 ), indicating that the procedure not only improved vision through refractive correction but also potentially enhanced visual system function by resolving factors like concomitant cataract.

This study revealed that Group A (AL < 16 mm) had high intraoperative and postoperative complication rates (33.3% and 66.7%, respectively), while Group C (AL 18–20 mm) had no complications, consistent with findings from larger studies by Day et al. [8] and Hammer et al. [27], confirming short AL as an independent risk factor.

The most common intraoperative complication was shallow AC/aqueous misdirection (20.0%), primarily occurring in eyes with AL < 18 mm. The mechanism involves the extremely small vitreous cavity volume in nanophthalmos, where anterior chamber opening can lead to forward movement of the anterior hyaloid face and lens-iris diaphragm, disrupting aqueous dynamics [14]. Therefore, advanced phaco platforms (e.g.2.2 mm micro-incision) and skilled phacoemulsification techniques are crucial for maintaining intraoperative anterior chamber stability [28].

The early postoperative complication rate was 40.0% (8/20), mainly shallow AC with elevated IOP (i.e., malignant glaucoma), accounting for 25.0% (5/20). The pathogenesis is considered a “vicious cycle” involving ciliary block, anterior hyaloid face block, and other factors [14]. While traditional medical and laser therapies have limited efficacy for such cases, aggressive intervention with pars plana vitrectomy (performed in 3 eyes in this study) achieved 100% success. This aligns with conclusions from Zhang et al. [21] in nanophthalmos, and is supported by recent evidence from Lincke et al. [29] showing that pars plana vitrectomy is effective for refractory aqueous misdirection.

### This study also explored the impact of other surgical details on safety

1. Clinical Significance of Prophylactic Peripheral Iridectomy (PI): Nanophthalmic eyes often exhibit significant postoperative inflammation, and adhesions between the iris and the implanted intraocular lens (IOL) were observed in a subset of patients. The literature widely attributes this to fragile blood-aqueous barrier function due to unique anatomy, leading to postoperative fibrin exudation and related synechiae [11], which is reported as a typical postoperative feature in nanophthalmos [4]. Notably, the newly created peripheral iridectomy (PI) itself carries a risk of inflammatory synechial or membranous closure, which can precipitate pupillary block and malignant glaucoma. Thus, the primary clinical value of the PI is to serve as a pre-established conduit for intervention in the event of postoperative shallow anterior chamber or malignant glaucoma. When ciliary block or IOL optic-posterior capsule adherence happens, the pupil often dilates poorly. Performing Nd: YAG laser posterior capsulotomy directly in the visual axis is not only technically challenging and risks IOL damage but also cannot ensure the newly created aqueous pathway effectively drains to the anterior chamber. In contrast, the PI area, even if membranous, is relatively easier to penetrate with Nd: YAG laser. This safely re-establishes communication between the posterior and anterior chambers, creating an aqueous drainage path that effectively bypasses the IOL haptics [29–31].
2. IOL Selection for Extremely Short Eyes (≤ 16 mm): Nanophthalmic patients often require high-power IOLs (+ 45 D to + 75 D), posing challenges in IOL selection and implantation technique [32]. To achieve emmetropia, piggyback IOL implantation (one in the bag, one in the sulcus) has been reported to provide sufficient refractive power [33]. However, caution is warranted as sulcus-placed IOLs can cause a range of anterior segment complications, such as iris pigment dispersion, iritis, peripheral anterior synechiae, and posterior synechiae [34].
  In this study, both patients receiving piggyback IOLs for emmetropia developed severe fibrinous exudation, shallow AC, and elevated IOP. One required emergency removal of the sulcus IOL on postoperative day 3 due to a very shallow AC with severe exudation, retaining the bag IOL, combined with anterior vitrectomy and repeat goniosynechialysis. The other, with uncontrolled IOP despite medical and cyclophotocoagulation therapy, accompanied by severe exudation and corneal edema, underwent secondary surgery (anterior vitrectomy + repeat goniosynechialysis) at 2 months. Fortunately, neither suffered irreversible optic nerve damage from persistent high IOP. These adverse events are consistent with documented complications of sulcus implantation [34].
  Based on our experience and literature evidence, we believe the primary surgical goal for extremely short eyes should be relieving anatomical obstruction and controlling IOP, placing refractive emmetropization as a secondary objective [3, 8]. Furthermore, studies indicate that existing IOL power calculation formulas are inaccurate in nanophthalmos, further reducing the reliability of achieving precise refractive targets [35]. Therefore, implanting a single IOL and correcting residual hyperopia with spectacles postoperatively is a safer, more reasonable, and practical choice [8]. Our analysis of 5 eyes with AL < 16 mm receiving single IOL implantation supports this strategy, showing little change in residual hyperopia compared to preoperative status (mean difference + 0.80 ± 1.48 D), which is usually well-tolerated by most long-term spectacle-wearing hyperopic patients.
3. Gonioscopic Synechialysis is Key to Successful IOP Control: This concept originates from the pioneering description of goniosynechialysis by Campbell and Vela [24], who emphasized that it must be performed under direct gonioscopic view to accurately separate synechiae and confirm successful re-exposure of the functional trabecular meshwork. Therefore, meticulous and extensive synechialysis, maximizing exposure of the functional trabecular meshwork, is the anatomical foundation for breaking aqueous outflow obstruction and achieving long-term stable IOP control.

## Conclusion

For nanophthalmos with secondary angle-closure glaucoma, Phaco + IOL+GSL appears to be a feasible and effective procedure in selected cases, particularly when combined with a stepwise complication management strategy. It may serve as an initial surgical option before resorting to more invasive combined procedures. However, the high complication rate in eyes with AL < 16 mm warrants extreme caution and readiness for escalation. The retrospective design and small sample size limit the generalizability of our findings; larger prospective studies are needed to confirm these results.

## Data Availability

The datasets used and/or analyzed during the current study are available from the corresponding author on reasonable request.

## Abbreviations

AC: Anterior Chamber
ACG: Angle-Closure Glaucoma
AL: Axial Length
BCVA: Best-Corrected Visual Acuity
IOL: Intraocular Lens
IOP: Intraocular Pressure
GSL: Goniosynechialysis
logMAR: Logarithm of the Minimum Angle of Resolution
PAC: Primary Angle Closure
PAS: Peripheral Anterior Synechiae
Phaco: Phacoemulsification
PI: Peripheral Iridectomy
PCR: Posterior Capsular Rupture
PPV: Pars Plana Vitrectomy
SE: Spherical Equivalent
UCVA: Uncorrected Visual Acuity

## References

[1] Singh OS, Simmons RJ, Brockhurst RJ, Trempe CL. Nanophthalmos: a perspective on identification and therapy. Ophthalmology. 1982;89(9):1006-12. PMID: 7177565. 7177565

[2] Luo NC, Zuo CG, Liu X. Research status and progress of nanophthalmos. Zhong hua Yan Ke Za Zhi. 2025;61(5):391–6. 10.3760/cma.j.cn112142-20240702-00289. 10.3760/cma.j.cn112142-20240702-00289 40302600

[3] Rajendrababu S, Shroff S, Uduman MS, Babu N. Clinical spectrum and treatment outcomes of patients with nanophthalmos. Eye (Lond). 2021;35(3):825–30. 10.1038/s41433-020-0971-4. 32461562 10.1038/s41433-020-0971-4 PMC8027642

[4] Wu W, Dawson DG, Sugar A, Elner SG, Meyer KA, McKey JB, Moroi SE. Cataract surgery in patients with nanophthalmos: results and complications. J Cataract Refract Surg. 2004;30(3):584 – 90. 10.1016/j.jcrs.2003.07.009. PMID: 15050253. 10.1016/j.jcrs.2003.07.009 15050253

[5] Yalvac IS, Satana B, Ozkan G, Eksioglu U, Duman S. Management of glaucoma in patients with nanophthalmos. Eye (Lond). 2008;22(6):838–43. 10.1038/sj.eye.6702742. 17293784 10.1038/sj.eye.6702742

[6] Abdelrahman AM, Ismail YM. Primary Transscleral Diode Laser Cyclophotocoagulation for Management of Glaucoma in Nanophthalmic Eyes. J Glaucoma. 2024;33(4):e15–8. 10.1097/IJG.0000000000002284. 37585376 10.1097/IJG.0000000000002284

[7] Aquino MC, Barton K, Tan AM, Sng C, Li X, Loon SC, Chew PT. Micropulse versus continuous wave transscleral diode cyclophotocoagulation in refractory glaucoma: a randomized exploratory study. Clin Exp Ophthalmol. 2015;43(1):40–6. 10.1111/ceo.12360. 24811050 10.1111/ceo.12360

[8] Day AC, MacLaren RE, Bunce C, Stevens JD, Foster PJ. Outcomes of phacoemulsification and intraocular lens implantation in microphthalmos and nanophthalmos. J Cataract Refract Surg. 2013;39(1):87–96. 10.1016/j.jcrs.2012.08.057. 23245362 10.1016/j.jcrs.2012.08.057

[9] Wei Y, Su Y, Fang L, Guo X, Chen S, Han Y, Zhu Y, Cheng B, Lin S, Zhong Y, Liu X. Effect of Combined Surgery in Patients with Complex Nanophthalmos. J Clin Med. 2022;11(19):5909. 10.3390/jcm11195909. 36233776 10.3390/jcm11195909 PMC9571930

[10] Fan X, Wang J, Sheng Q, Zhai R, Kong X. Outcomes of combined phacoemulsification, anterior vitrectomy, and sclerectomy in nanophthalmic eyes with glaucoma. Eye (Lond). 2023;37(4):751–9. 10.1038/s41433-022-02039-w. Epub 2022 Apr 5. PMID: 35383309; PMCID: PMC9998427. 35383309 10.1038/s41433-022-02039-w PMC9998427

[11] He MY, Feng JR, Zhang L. Treatment of Nanophthalmos Cataracts: Surgery and Complications. Semin Ophthalmol. 2022;37(7–8):849–55. 10.1080/08820538.2022.2102929. 35856463 10.1080/08820538.2022.2102929

[12] Tang YH, Li YY, Xie YQ, Zhang SD, Xu SX, Le RR, Chu XZ, Hu CJ, Wang XJ, Pan WH, Liang YB. Phacoemulsification combined with goniosynechialysis under a gonioscope in the treatment of angle-closure glaucoma: the effect of reopening the synechial anterior chamber angle. Zhonghua Yan Ke Za Zhi. 2022;58(9):701–5. 10.3760/cma.j.cn112142-20211127-00565. 36069091 10.3760/cma.j.cn112142-20211127-00565

[13] Zhao J, Zhang C, Pazo EE, Dai G, Li Y, Chen Y, Li M, Che H. Phaco-goniosynechialysis versus phaco-trabeculectomy in patients with refractory primary angle-closure glaucoma: a comparative study. BMC Ophthalmol. 2023;23(1):144. 10.1186/s12886-023-02885-6. 37024836 10.1186/s12886-023-02885-6 PMC10080734

[14] Quigley HA, Friedman DS, Congdon NG. Possible mechanisms of primary angle-closure and malignant glaucoma. J Glaucoma. 2003;12(2):167–80. 10.1097/00061198-200304000-00013. 12671473 10.1097/00061198-200304000-00013

[15] Lam AK, Chan R, Pang PC. The repeatability and accuracy of axial length and anterior chamber depth measurements from the IOLMaster. Ophthalmic Physiol Opt. 2001;21(6):477 – 83. 10.1046/j.1475-1313.2001.00611.x. PMID: 11727876. 10.1046/j.1475-1313.2001.00611.x 11727876

[16] Yang M, Pan X, Chen Z, Shen X, Yu Z, Tao Y, Li S, Mo X, Liu X, Fan N. Management of Pseudophakic Malignant Glaucoma Using Modified Nd:YAG Laser Treatment Methodology Through Surgical Preset Iridectomy. Ophthalmol Ther. 2024;13(1):337–51. 10.1007/s40123-023-00851-5. Epub 2023 Nov 20. PMID: 37982983; PMCID: PMC10776534. 37982983 10.1007/s40123-023-00851-5 PMC10776534

[17] Holladay JT. Proper method for calculating average visual acuity. J Refract Surg. 1997;13(4):388–91. 10.3928/1081-597X-19970701-16. 9268940 10.3928/1081-597X-19970701-16

[18] Nossair AA, Ewais WA, Ali LS. Retrospective Study of Vitreous Tap Technique Using Needle Aspiration for Management of Shallow Anterior Chamber during Phacoemulsification. J Ophthalmol. 2017;2017:2801025. 10.1155/2017/2801025. Epub 2017 May 9. PMID: 28607771; PMCID: PMC5451757. 28607771 10.1155/2017/2801025 PMC5451757

[19] Kuriakose T, Jasper S, Thomas S. Pars-plana fluid aspiration for positive vitreous cavity pressure in anterior segment surgeries. Indian J Ophthalmol. 2018;66(4):565–7. 10.4103/ijo.IJO_939_17. PMID: 29582821; PMCID: PMC5892063. 29582821 10.4103/ijo.IJO_939_17 PMC5892063

[20] Yao L, Wang H, Wang Y, Zhao P, Bai H. Phacoemulsification combined with goniosynechialysis versus phacoemulsification alone for patients with primary angle-closure disease: A meta-analysis. PLoS ONE. 2024;19(2):e0296849. 10.1371/journal.pone.0296849. PMID: 38306318; PMCID: PMC10836673. 38306318 10.1371/journal.pone.0296849 PMC10836673

[21] Zhang Z, Zhang S, Jiang X, Wei Y. Combined 23-G Pars Plana Vitrectomy and Lensectomy in the Management of Glaucoma Associated with Nanophthalmos. Ophthalmic Res. 2018;59(1):37–44. 10.1159/000477620. 28719889 10.1159/000477620

[22] Rajendrababu S, Babu N, Sinha S, Balakrishnan V, Vardhan A, Puthuran GV, Ramulu PY. A Randomized Controlled Trial Comparing Outcomes of Cataract Surgery in Nanophthalmos With and Without Prophylactic Sclerostomy. Am J Ophthalmol. 2017;183:125–33. 10.1016/j.ajo.2017.09.008. 28911991 10.1016/j.ajo.2017.09.008

[23] Wang XJ, Tian AJ, Zhang SD, et al. Multicenter retrospective analysis of treatment methods for acute primary angle-closure glaucoma in hospitalized patients. Zhonghua Yan Ke Za Zhi. 2025;61(09):667–76. 10.3760/cma.j.cn112142-20241031-00490. 40921656 10.3760/cma.j.cn112142-20241031-00490

[24] Campbell DG, Vela A. Modern goniosynechialysis for the treatment of synechial angle-closure glaucoma. Ophthalmology. 1984;91(9):1052-60. 10.1016/s0161-6420(84)34195-1. PMID: 6493714. 10.1016/s0161-6420(84)34195-1 6493714

[25] Miyako F, Hirooka K, Onoe H, Kiuchi Y. Risk factors for transient ciliochoroidal detachment after goniotomy with the Kahook Dual Blade. Sci Rep. 2024;14(1):21725. 10.1038/s41598-024-72715-2. PMID: 39289459; PMCID: PMC11408665. 39289459 10.1038/s41598-024-72715-2 PMC11408665

[26] Sethi A, Udenia H, Beri N, Angmo D, Bari A, Sharma N, Dada T. Surgical management of cyclodialysis cleft: an update. J Curr Glaucoma Pract. 2025 Jul-Sep;19(3):143–52. 10.5005/jp-journals-10078-1469. Epub 2025 Oct 4. PMID: 41113793; PMCID: PMC12533715. 10.5005/jp-journals-10078-1469 PMC12533715 41113793

[27] Hammer M, Teich L, Friedrich M, Reitemeyer E, Britz L, Khoramnia R, Yildirim TM, Auffarth GU. Cataract surgery in the extremely small eye: morphology, comorbidities and outcomes in 300 eyes. Br J Ophthalmol. 2025;109(8):917–24. 10.1136/bjo-2024-326998. 40015941 10.1136/bjo-2024-326998 PMC12320606

[28] Kim JY, Kim H, Jun I, Kim TI, Seo KY. Effect and Safety of Pressure Sensor-equipped Handpiece in Phacoemulsification System. Korean J Ophthalmol. 2023;37(5):387–94. 10.3341/kjo.2022.0157. 37562441 10.3341/kjo.2022.0157 PMC10587456

[29] Lincke JB, Häner N, Schawkat M, Zinkernagel MS, Unterlauft JD. Treatment of pseudophakic aqueous misdirection syndrome. Sci Rep. 2025;15(1):1415. 10.1038/s41598-024-83659-y. PMID: 39788995; PMCID: PMC11718072. 39788995 10.1038/s41598-024-83659-y PMC11718072

[30] Safir M, Hecht I, Sharon T, Einan-Lifshitz A, Belkin A. Application of Nd:YAG laser to the anterior vitreous in malignant glaucoma - a systemic review and meta-analysis. Graefes Arch Clin Exp Ophthalmol. 2022;260(9):2981–90. 10.1007/s00417-022-05640-7. Epub 2022 Mar 29. PMID: 35348842. 35348842 10.1007/s00417-022-05640-7

[31] Schmidt DC, Kessel L, Pedersen KB, Villumsen JE, Bach-Holm D. Pars plana vitrectomy combined with hyaloido-zonula-iridectomy in treatment of patients with chronic aqueous misdirection: A systematic literature review and case series. Acta Ophthalmol. 2021;99(3):251–9. 10.1111/aos.14580. Epub 2020 Aug 25. PMID: 32840056. 32840056 10.1111/aos.14580

[32] Yosar JC, Zagora SL, Grigg JR. Cataract Surgery in Short Eyes, Including Nanophthalmos: Visual Outcomes, Complications and Refractive Results. Clin Ophthalmol. 2021;15:4543–51. 10.2147/OPTH.S344465. 34866899 10.2147/OPTH.S344465 PMC8636843

[33] Cao KY, Sit M, Braga-Mele R. Primary piggyback implantation of 3 intraocular lenses in nanophthalmos. J Cataract Refract Surg. 2007;33(4):727–30. 10.1016/j.jcrs.2006.11.028. 17397750 10.1016/j.jcrs.2006.11.028

[34] LeBoyer RM, Werner L, Snyder ME, Mamalis N, Riemann CD, Augsberger JJ. Acute haptic-induced ciliary sulcus irritation associated with single-piece AcrySof intraocular lenses. J Cataract Refract Surg. 2005;31(7):1421–7. 10.1016/j.jcrs.2004.12.056. 16105617 10.1016/j.jcrs.2004.12.056

[35] Moore JE, McNeely RN, Moutari S. Cataract surgery in the small adult eye: a review. Clin Exp Ophthalmol. 2025;53(5):558–569. 10.1111/ceo.14510. Epub 2025 Mar 4. PMID: 40035171. 10.1111/ceo.14510 40035171

